# A study on possible use of *Urtica dioica* (common nettle) plants as uranium (^234^U, ^238^U) contamination bioindicator near phosphogypsum stockpile

**DOI:** 10.1007/s10967-015-4302-3

**Published:** 2015-07-26

**Authors:** Grzegorz Olszewski, Alicja Boryło, Bogdan Skwarzec

**Affiliations:** Laboratory of Analytical and Environmental Radiochemistry, Department of Environmental Chemistry and Radiochemistry, Faculty of Chemistry, University of Gdańsk, 80-308 Gdańsk, Poland

**Keywords:** *Urtica dioica*, Uranium, Phosphogypsum, Translocation factor, Bioaccumulation factor, ^234^U/^238^U

## Abstract

The aim of this study was to determine uranium concentrations in common nettle (*Urtica dioica*) plants and corresponding soils samples which were collected from the area of phosphogypsum stockpile in Wiślinka (northern Poland). The uranium concentrations in roots depended on its concentrations in soils. Calculated BCF and TF values showed that soils characteristics and air deposition affect uranium absorption and that different uranium species have different affinities to *U*. *dioica* plants. The values of ^234^U/^238^U activity ratio indicate natural origin of these radioisotopes in analyzed plants. Uranium concentration in plants roots is negatively weakly correlated with distance from phosphogypsum stockpile.

## Introduction

Phosphogypsum is a byproduct from phosphoric acid production from phosphate rocks usually stored in specially designated areas. The phosphogypsum stockpile in Wiślinka (northern Poland) is located between the Martwa Wisła river and farm fields, close to the Gdańsk agglomeration. It is considered to be one of the major contaminators of Vistula river delta. The stockpile contains about 16 million tons of phosphogypsum. Phosphate rocks used for phosphoric acid production are characterized by high content of natural alpha radioactive elements, especially from uranium decay series (^210^Po, ^226^Ra, ^234^U, ^238^U) and beta emitter (^210^Pb). In the process of phosphoric acid production about 80 % of uranium is associated with the phosphoric acid fraction, while about 90 % of the ^210^Po and ^210^Pb is bound to the phosphogypsum fraction [1–3]. Phosphogypsum in Wiślinka might have serious radiological impact on the local environment. Radionuclides might be leached by wet precipitation and transported through groundwaters to plants where they are accumulated [4–9].

Natural uranium consists of three alpha radioactive isotopes: 99.2745 % of ^238^U, 0.7200 % of ^235^U, and 0.0054 % of ^234^U [10]. Environmental occurrence of uranium can be a result of human activities. As a major uranium sources in the environment can be considered nuclear industry, combustion of fossil fuels, production and use of phosphorous fertilizers or use of depleted uranium for military purposes [11–13]. Normally in water ^234^U and ^238^U radionuclides are not in the radioactive state of equilibrium. In groundwaters, the average values of the activity ratio between ^234^U and ^238^U are in the range from 0.51 to 9.02, in salt water from 1.11 to 5.14, in river water from 1.00 to 2.14, in river suspension from 0.80 to 1.00, in oceanic water 1.14 and in Baltic water 1.17 [14–16]. In rocks, soils and sediments the uranium isotopes ^234^U and ^238^U are in relative equilibrium (from 0.84 to 1.19 for oceanic basalts, from 0.70 to 1.16 for phosphorite concretions, from 0.83 to 1.28 for oceanic sediments and from 0.98 to 1.04 for Baltic sediments) [17, 18].

The main factors that contribute to uranium content and leaching ability in soil environment are the proximity of the water to the uranium source, the degree of hydraulic isolation of the water from dilution by fresher water, climatic effects and their seasonal variability (evapotranspiration), the pH and *E*_h_ of the water (uranium is mobilized under oxidizing conditions and immobilized under reducing conditions), concentrations of important species that can form either strong complexes or precipitate insoluble uranium minerals (e.g., carbonate, phosphate, vanadate, fluoride, sulfate, silicate, calcium, potassium) and the presence of highly sorbents like organic matter or Fe/Mn/Ti oxyhydroxides [19, 20]. In case of plants large number of factors control metal accumulation and bioavailability such as soil and climatic conditions, plant genotype and agronomic management, including: active/passive transfer processes, sequestration and speciation, redox states, the type of plant root system and the response of plants to elements in relation to seasonal cycles [21, 22]. Structure of the soil is also considered as one of the major factors that contribute to extent of the metals taken up by the plants. Such factors as clay particles, metal solubility controlled by pH, amount of metals cations exchange capacity, organic carbon content and oxidation state of the system are also important in metals availability [22].

The main aim of this work was to establish a possible use of *Urtica dioica* (common nettle) plants as uranium contamination bioindicator in the area of phosphogypsum stockpile by analysis of ^234^U and ^238^U in plants samples and corresponding soils and to examine the impact of phosphogypsum stockpile on the surrounding environment. Additionally, the values of the ^234^U/^238^U activity ratio and BCF (biocencentration) and TF (translocation) factors are calculated in order to define both the possible uranium sources and level of their accumulation in plants.

## Experimental

### Collection of samples

**Fig. 1:**
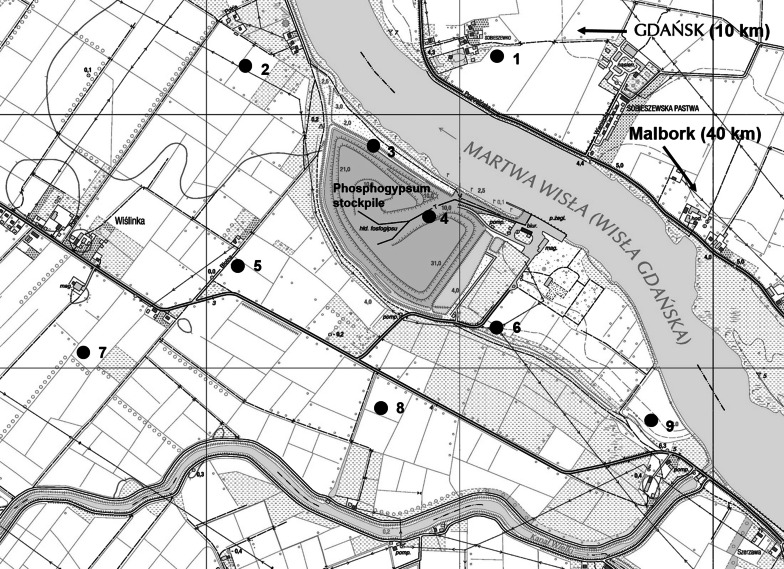
Sample collection sites

The *U*. *dioica* plants samples along with corresponding soils were collected from the area of phosphogypsum stockpile in Wiślinka (northern Poland) in October 2013. The locations of the analyzed plants and soils samples are presented in Fig. 1. Control samples were collected in Malbork (Pomeranian Voivodeship). As the aim of the study was to collect multiple samples in various distances from stockpile, *U*. *dioica* due to it commonness seemed to be the most appropriate plant.

### Preparation of samples

Collected plants were divided into green part and root. Roots were washed with double deionized water in order to remove soil particles. Green parts were cleaned from soils particles but not washed in order to retain possible aerial contamination (wet and dry deposition and phosphogypsum particles). Before analysis, each sample was air dried, homogenized using mortar and dried in 60 °C. Additionally, soils samples were passed through 0.25 mm sieve. From homogenized sample three subsamples were weighted and enriched with approximately 15 mBq (plants) and 30 mBq (soils) of ^232^U as the yield tracer.

### Analysis of samples

#### Radioanalytical proceducre

The analytical procedure of determination of uranium radioisotopes (^234^U, ^238^U) in analyzed samples was based on the mineralization of soils samples in concentrated acids HNO_3_ and HCl, mineralization of plants samples in concentrated HNO_3_ acid with H_2_O_2_ addition and separation on the anion exchange resins according to method established by Skwarzec and Boryło [23, 24].

#### Instrument for analysis

The activities of ^234^U and ^238^U were measured using alpha spectrometer (Alpha Analyst S470) equipped with a surface barrier PIPS detector with an active surface of 300 mm^2^ placed in a vacuum chamber connected to a 1024 multichannel analyzer (Canberra–Packard, USA). Detector yield ranged from 0.30 to 0.40. In most of the used detectors with a surface of 300 mm^2^, the resolution was 17–18 keV. Minimal detectable activity (MDA) was measured to be 0.2 mBq for both ^238^U and ^234^U [25]. The results of ^234^U and ^238^U concentrations in analyzed samples are given with expanded standard uncertainty calculated for a 95 % CI. The concentrations of uranium isotopes in the IAEA-330 and IAEA-375 samples were consistent with the reference values reported by the IAEA. The accuracies for ^234^U, ^238^U determinations were high as all analyzed values were within certified reference confidence intervals with precisions estimated to be less than 5 %. Bioconcentration factor (BCF) and translocation factors (TF) were calculated as [7]:

1 $$ {\text{BCF}} = \frac{{{\text{Concentration}}_{\text{root}} }}{{{\text{Concentration}}_{\text{soil}} }} $$

2 $$ {\text{TF}}_{{\frac{\text{green\;part}}{\text{soil}}}} = \frac{{{\text{Concentration}}_{\text{green\;part}} }}{{{\text{Concentration}}_{\text{soil}} }} $$

3 $$ {\text{BCF}}_{{\frac{\text{plant}}{\text{soil}}}} = \frac{{{\text{Concentration}}_{\text{plant}} }}{{{\text{Concentration}}_{\text{soil}} }} $$

4 $$ {\text{TF}} = \frac{{{\text{Concentration}}_{\text{green\;part}} }}{{{\text{Concentration}}_{\text{root}} }} $$

## Results and discussion

### ^238^U, ^234^U and total uranium concentration in *Urtica dioica* roots, shoots and soils

The concentrations of ^234^U and ^238^U radioisotopes in analyzed plants and soils samples are given in Table 1. ^238^U concentrations ranged from 0.05 ± 0.03 to 2.46 ± 0.11 mBq g^−1^ dry wt in green parts of analyzed *U*. *dioica*, from 0.15 ± 0.06 to 2.50 ± 0.03 mBq g^−1^ dry wt in roots and from 6.0 ± 0.6 to 29.3 ± 1.5 mBq g^−1^ dry wt in corresponding soils samples. For analyzed control sample the obtained results for ^238^U are 1.00 ± 0.04 mBq g^−1^ dry wt in green part, 1.03 ± 0.15 mBq g^−1^ dry wt in root and 5.9 ± 0.4 mBq g^−1^ dry wt for soil. Total uranium values calculated for green parts, roots, whole plants of analyzed *U*. *dioica* plants and corresponding soils are presented in Table 2.

**Table 1:** Average ^238^U, ^234^U concentration and ^234^U/^238^U activity ratio in analyzed *Urtica dioica* plants (given with expanded standard uncertainty calculated for 95 % CI)

Sample collection site	^238^U concentration (mBq g^−1^ dry wt)			^234^U concentration (mBq g^−1^ dry wt)			^234^U/^238^U activity ratio		
	Green part	Root	Soil	Green part	Root	Soil	Green part	Root	Soil
1	0.39 ± 0.05	0.61 ± 0.04	9.2 ± 0.7	0.44 ± 0.04	0.65 ± 0.03	9.3 ± 0.5	1.13 ± 0.21	1.06 ± 0.09	1.00 ± 0.08
2	0.48 ± 0.08	1.48 ± 0.09	6.0 ± 0.6	0.54 ± 0.09	1.46 ± 0.12	5.9 ± 0.6	1.12 ± 0.11	1.02 ± 0.12	1.00 ± 0.06
3	0.05 ± 0.03	2.50 ± 0.03	29.3 ± 1.5	0.06 ± 0.03	2.78 ± 0.12	30.4 ± 1.2	1.29 ± 0.72	1.11 ± 0.06	1.04 ± 0.01
4	0.07 ± 0.05	1.89 ± 0.07	28.6 ± 1.7	0.08 ± 0.03	2.48 ± 0.15	30.8 ± 2.9	1.17 ± 0.87	1.31 ± 0.15	1.08 ± 0.04
5	1.26 ± 0.07	0.39 ± 0.04	11.3 ± 0.7	1.06 ± 0.04	0.39 ± 0.01	12.5 ± 0.5	0.84 ± 0.05	1.03 ± 0.13	1.10 ± 0.05
6	1.46 ± 0.10	1.07 ± 0.10	15.7 ± 0.8	1.51 ± 0.02	1.15 ± 0.12	16.6 ± 0.9	1.03 ± 0.06	1.07 ± 0.08	1.06 ± 0.11
7	0.65 ± 0.06	1.22 ± 0.05	8.9 ± 0.5	0.76 ± 0.08	1.02 ± 0.16	8.6 ± 0.7	1.18 ± 0.09	0.83 ± 0.15	0.96 ± 0.12
8	2.46 ± 0.11	0.78 ± 0.04	20.4 ± 1.0	2.23 ± 0.13	0.82 ± 0.06	20.7 ± 1.4	0.91 ± 0.10	1.06 ± 0.05	1.01 ± 0.07
9	0.22 ± 0.06	0.15 ± 0.06	3.7 ± 0.4	0.25 ± 0.07	0.17 ± 0.05	4.3 ± 0.3	1.09 ± 0.15	1.11 ± 0.11	1.17 ± 0.08
Control sample	1.00 ± 0.04	1.03 ± 0.15	5.9 ± 0.4	1.00 ± 0.11	1.11 ± 0.28	6.6 ± 0.2	1.00 ± 0.09	1.07 ± 0.11	1.11 ± 0.04

**Table 2:** Average total uranium concentration in analyzed *Urtica dioica* plants (given with expanded standard uncertainty calculated for 95 % CI)

Sample collection site	Total uranium (mg kg^−1^ dry wt)			
	Green part	Root	Total plant	Soil
1	0.032 ± 0.004	0.050 ± 0.003	0.082 ± 0.005	0.753 ± 0.056
2	0.039 ± 0.007	0.121 ± 0.008	0.160 ± 0.010	0.486 ± 0.049
3	0.004 ± 0.003	0.204 ± 0.002	0.208 ± 0.003	2.390 ± 0.040
4	0.006 ± 0.004	0.155 ± 0.005	0.161 ± 0.007	2.330 ± 0.060
5	0.103 ± 0.006	0.033 ± 0.006	0.136 ± 0.008	0.924 ± 0.058
6	0.119 ± 0.008	0.088 ± 0.008	0.207 ± 0.012	1.281 ± 0.068
7	0.053 ± 0.005	0.099 ± 0.002	0.152 ± 0.005	0.727 ± 0.042
8	0.201 ± 0.009	0.063 ± 0.003	0.264 ± 0.010	1.662 ± 0.084
9	0.019 ± 0.005	0.012 ± 0.005	0.031 ± 0.007	0.299 ± 0.034
Control sample	0.082 ± 0.003	0.084 ± 0.012	0.166 ± 0.013	0.484 ± 0.029

#### Comparison with other studies on uranium uptake by plants

**Fig. 2:**
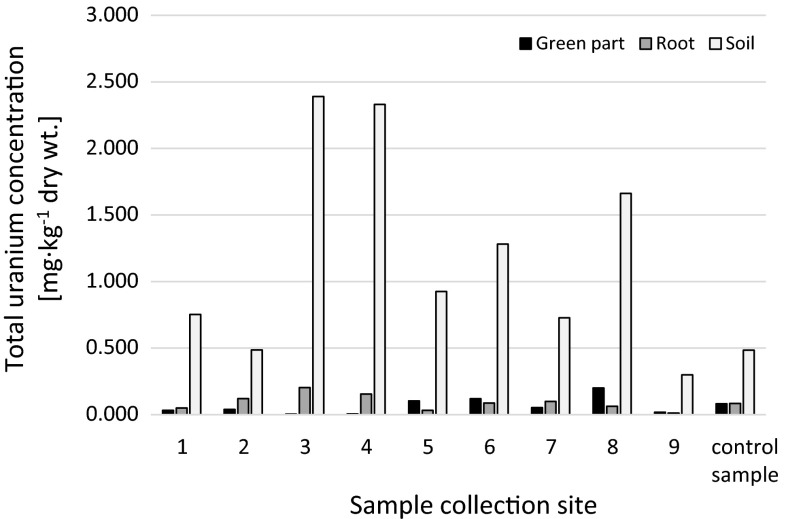
Total uranium concentration in analyzed *Urtica dioica* and soils samples

Wild and cultivated plants from the immediate vicinity of uranium waste dumps in Ronneburg in Germany stored normal to eightfold uranium contents. Leafy plant species accumulated much uranium, whereas tubes, thick parts of stalks, fruits and grains stored less uranium. With increasing age of the vegetation uranium content decreased significantly [26]. The obtained results for *U*. *dioica* samples were similar or insignificantly higher in comparison to other results. Uranium in lettuce plants growing in contaminated soils (near uranium mining facilities in Portugal) ranged from 0.95 to 6 mg kg^−1^ dry wt in roots and from 0.32 to 2.6 mg kg^−1^ dry wt in leaves [27]. The concentration of uranium in shoots of plant species grown in soil contaminated with 100 mg kg^−1^ of uranium ranged between 3.2 and 24 mg kg^−1^ dry wt Sunflower and Indian mustard had the highest uranium concentrations in shoots (24.6 and 21.8 mg kg^−1^ dry wt, respectfully), while wheat and ryegrass had the lowest concentrations (3.2 and 3.8 mg kg^−1^ dry wt). On the other hand, uranium concentrations in roots of the analyzed plants were significantly higher than in shoots and varied from 89 to 810 mg kg^−1^ dry wt what were 30–50 times greater than its concentration in shoots [28]. Al-Kharouf et al. measured concentrations of ^234^U and ^238^U in watermelon and zucchini crops harvested on irrigated cultivated area which lies above superficial uranium deposits. The average ^234^U and ^238^U concentrations were found to be 0.017 and 0.010 mBq g^−1^ dry wt, respectively. ^234^U and ^238^U concentrations in watermelon green parts with roots had average of 0.81 and 0.65 mBq g^−1^ dry wt respectively, what was an order of magnitude higher than in pulp. Zucchini fruits had concentrations below the detection limit of 1 × 10^−4^ mBq g^−1^ dry wt for ^234^U and ^238^U. The average ^234^U and ^238^U concentrations in zucchini green parts with roots were 0.75 ± 0.04 and 0.72 ± 0.03 mBq g^−1^ dry wt, respectively [29]. The highest uranium concentrations were measured in soils, different crops and vegetables from the uranium mining area in Jiangxi province in southeastern China: 3159 ± 415 mBq g^−1^ dry wt for soil samples, while the mean specific activities of ^238^U in analyzed plants ranged from 15 to 118 and from 108 to 1167 mBq g^−1^ dry wt for the shoots and roots, respectively [30]. In 2011 series of different plants (meadow, hygrophilous, edible, ruderal plants and corn) were collected around phosphogypsum stockpile in Wiślinka and surveyed on uranium contents. Total uranium concentrations depended on plant type. The uranium content was associated with the plants age, root system (e.g. storage root system, taproot system, superior root system, and fibrous root system) and plant tomentose [7]. Other authors also show that uranium is accumulated in plants depending on their species and cultivars [31, 32]. In case of *U*. *dioica* it is clearly seen that localization has a crucial impact on uranium accumulation. Most of the authors report that higher uranium concentrations are present in root system of plants [28, 31, 33, 34]. This aspect is not so obvious in our research as there are samples with higher uranium concentrations in above-ground parts (Fig. 2).

### Impact of the phosphogypsum stack

**Fig. 3:**
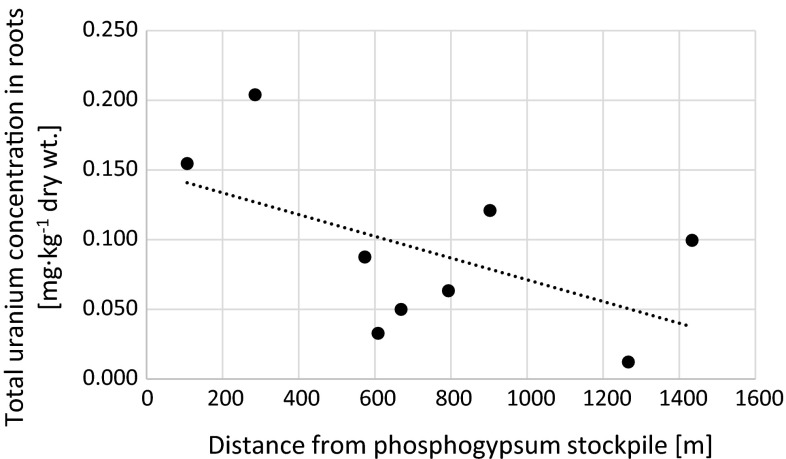
Relation between total uranium concentration in roots of analyzed *Urtica dioica* plants and distance from the phosphogypsum stockpile (*r*_s_ = −0.43)

**Fig. 4:**
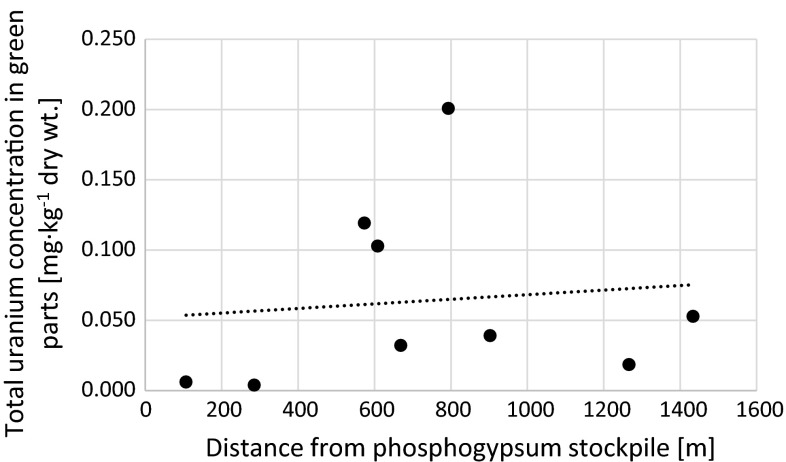
Relation between total uranium concentration in green parts of analyzed *Urtica dioica* plants and distance from the phosphogypsum stockpile (*r*_s_ = −0.30)

**Fig. 5:**
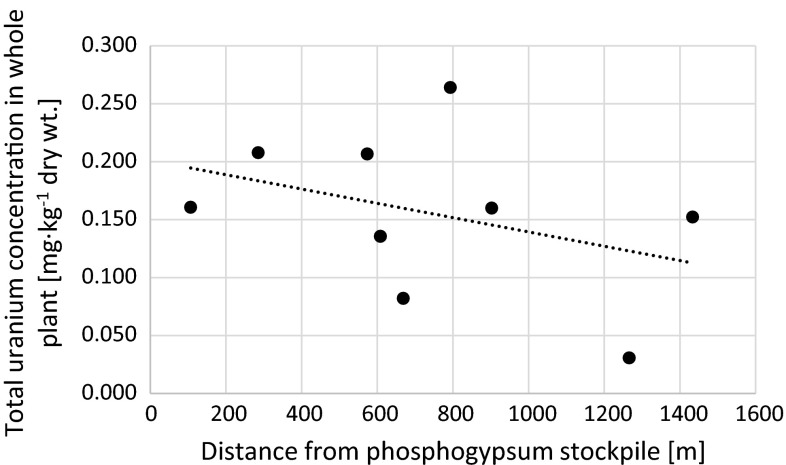
Relation between total uranium concentration in whole analyzed *Urtica dioica* plants and distance from the phosphogypsum stockpile (*r*_s_ = −0.45)

Possible impact of uranium from phosphogypsum stockpile on green parts, roots and whole plants of *U*. *dioica* in respect with distance from the stockpile was evaluated. The results revealed that total uranium concentrations in analyzed roots are weakly but negatively correlated with distance (*r*_s_ = −0.43) (Fig. 3). Similar effect can be noticed for uranium concentrations in analyzed soils samples (*r*_s_ = −0.83). This is not a general trend in this area. Some of the previously analyzed soils samples contained higher uranium concentrations than phosphogypsum what can be explained by the use of phosphate fertilizers in this area [35]. Opposite effect can be noticed in green parts. One of our main aims was to evaluate possible green parts contamination with phosphogypsum dust. There is no correlation between total uranium concentration in shoots and distance from phosphypsum stockpile (Fig. 4) and slight correlation between whole *U*. *dioica* plants and distance (Fig. 5). Only three samples (5, 6 and 8) contained increased uranium concentrations in shoots (Figs. 2 and 4). These three sites are located in open area that can be affected by air deposition with possible phosphogypsum particles. In 2012 wind directions in the area of Wiślinka were examined. The dominant winds were southern, northern and western [36]. The possibility of air transportation of both phosphogypsum and sewage sludge particles that cover the stockpile cannot be neglected. This fact may be connected with the northern-western wind that was observed in this area.

### The values of ^234^U/^238^U activity ratios

The values of ^234^U/^238^U activity ratio in analyzed environmental samples ranged between 0.84 ± 0.05 and 1.29 ± 0.72 for green parts, 0.83 ± 0.15 and 1.31 ± 0.15 for roots as well as 0.96 ± 0.12 and 1.17 ± 0.08 in corresponding soils (Table 1). The obtained values of the ^234^U/^238^U activity ratios for *U*. *dioica* samples are typical for plants, where the variations lie between 1.02 and 1.30 [7, 14]. Higher values of this activity ratio might be connected with plants interaction with water. The plants could have higher values of ^234^U/^238^U activity ratios than sediments suggesting that main source of uranium is water [37]. The similar effect was observed in this area in previous years [6]. Typical value of ^234^U/^238^U activity ratio for soils lies between 0.5 and 1.3 and is dependent on the geological surface [38]. Activity ratios obtained for soils in our research are typical for terrestrial environment. As a confirmation we calculated Spearman correlation factors for ^234^U and ^238^U concentrations in green parts and roots of *U*. *dioica* as well as soils. We received statistically significant *r*_s_ values: 1.00 for green parts, 0.98 for roots and 0.98 for soils. Spearman’s rank correlation is a non-parametrical alternative for Pearson’s correlation. It can be used to calculate the correlation between two variables that do not have normal distribution and are not linear. What is more, Spearman’s rank correlation is resistant for outlier results [39].

### The values of uranium BCF and TF in *Urtica dioica*

In order to understand the aspects of uranium phytoaccumulation in analyzed *U*. *dioica* samples we calculated TF, TF_green part/soil_ and BCF, BCF_plant/soil_ according to Eqs. (1)–(4). The obtained factors are presented in Table 3. Depending on the sample collection site these factors ranged from 0.035 ± 0.004 to 0.249 ± 0.010 for BCF, from 0.002 ± 0.001 to 0.121 ± 0.008 for TF_green part/soil_, from 0.069 ± 0.045 to 0.329 ± 0.068 for BCF_plant/soil_ and from 0.02 ± 0.01 to 3.22 ± 0.08 for TF. In control samples obtained BCF and TF are 0.97 ± 0.06, 0.174 ± 0.005, 0.168 ± 0.007 and 0.342 ± 0.008, respectively. The highest BCF factor observed for sample number 2 was more than seven times higher than the lowest for sample number 5.

**Table 3:** Average values of calculated BCF and TFs factors (given with combined standard uncertainty)

Sample collection site	TF	BCF	TF_green part/soil_	BCF_plant/soil_
1	0.64 ± 0.09	0.066 ± 0.007	0.043 ± 0.006	0.109 ± 0.017
2	0.32 ± 0.06	0.249 ± 0.010	0.080 ± 0.016	0.329 ± 0.068
3	0.02 ± 0.01	0.085 ± 0.004	0.002 ± 0.001	0.087 ± 0.056
4	0.04 ± 0.03	0.066 ± 0.005	0.003 ± 0.002	0.069 ± 0.045
5	3.22 ± 0.08	0.035 ± 0.004	0.111 ± 0.009	0.147 ± 0.030
6	1.36 ± 0.16	0.068 ± 0.007	0.093 ± 0.008	0.161 ± 0.021
7	0.53 ± 0.05	0.137 ± 0.008	0.073 ± 0.008	0.209 ± 0.023
8	3.17 ± 0.22	0.038 ± 0.003	0.121 ± 0.008	0.159 ± 0.013
9	1.52 ± 0.13	0.041 ± 0.017	0.062 ± 0.018	0.103 ± 0.051
Control sample	0.97 ± 0.06	0.174 ± 0.005	0.168 ± 0.012	0.342 ± 0.056

#### Comparison with other uranium TF and BCF studies

The obtained TF_green part/soil_ values for uranium are slightly different than reported in other studies. TF_green part/soil_ for lettuce lied between 0.011 and 0.023 [27], while for zucchini and watermelon was 4.21 × 10^−2^ and 1.82 × 10^−2^ [29]. TF_green part/soil_ and BCF values of vegetables grown in soils affected by uranium mining ranged from 0.005 to 0.037 and from 0.042 to 0.39, respectively [30]. Manigandan and Manikandan reported that uranium uptake by plants is low and BCF_plant/soil_ ratios were between 0.303 and 0.354 for different plant species [40]. Al-Masri et al. observed that vegetables characterized with relatively higher TF_green part/soil_ than their fruits [41]. Vera Tome et al. reported BCF_plant/soil_ for ^238^U in range of 0.020–0.250 what is similar to our results [42]. Sheppard et al. studied uptake of natural radionuclides by field and garden crops and reported an overall geometric mean BCF_plant/soil_ of 0.013 for uranium [43] while IAEA reports overall range of 10^−2^–10^−4^ [44]. In 2005 Sheppard et al. published results for uranium BCF_plant/soil_ values in plants from the area of uranium refinery and background sites across the Canada. The average BCF_plant/soil_ were 0.0068 and 0.0035, respectively [45]. BCF and TFs values received for control sample from Malbork suggest that these values are rather dependent on soils characteristics and uranium bioavailability. For analyzed meadow, hygrophilous, edible, ruderal and corn plants we observed different BCF and similar TF values (TF were in range of 0.05 for edible plants to 0.86 for hygrophilous plants, while BCF values ranged from 0.54 to 1.63). BCF values differences can be explained by different solum, substratum and bioavailability of uranium [7]. TF values were similar but no factors higher than 1 were noticed. The differences between TF values are probably connected with wet and dry air deposition and different tomentose that is dependent on plant type [7]. The comparison between BCF and TF values for *U*. *dioica* and other plants is presented on Table 4.

**Table 4:** A comparison between obtained BCF and TF values in *Urtica dioica* and other plants

Plant	BCF	BCF_plant/soil_	TF	TF_green part/soil_	References
Edible plants	1.63		0.05		[7]
Hygrophilous	0.80		0.86		
Corn	0.58		0.11		
Ruderal plants	0.54		0.20		
Meadow plants	1.5		0.11		
Lettuce				0.011–0.023	[27]
Watermelon				0.018	[29]
Zucchini				0.042	
Vegetables	0.042–0.39			0.005–0.037	[30]
Wild plants		0.303–0.354			[40]
Crops				0.036–0.059	[41]
Grass		0.020–0.250			[42]
Garden crops		0013			[43]
plants		0.001–0.1			[44]
Wheat	0.9			0.03	
Sunflower	8.1			0.38	[28]
Switchgrass	1.9			0.06	
Nettle	0.035–0.249	0.069–0.342	0.02–3.22	0.002–0.168	This study

#### TF and BCF values variability explanation

**Fig. 6:**
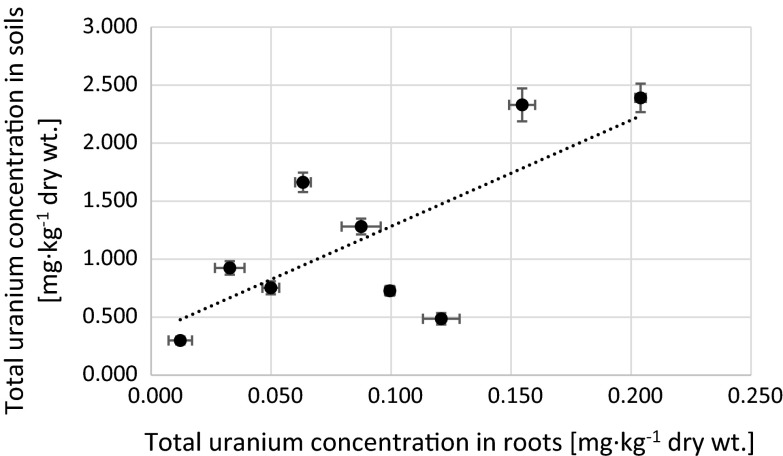
Correlation between total uranium concentration in roots and soils corresponding to analyzed *Urtica dioica* samples (*r*_s_ = 0.55)

**Fig. 7:**
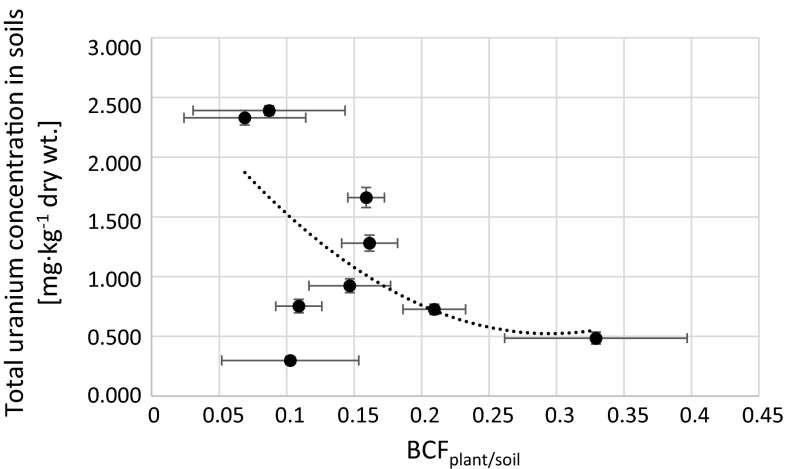
Relation between calculated BCF_plant/soil_ and total uranium concentration in soils corresponding to analyzed *Urtica dioica* plants (*r*_s_ = −0.48)

**Fig. 8:**
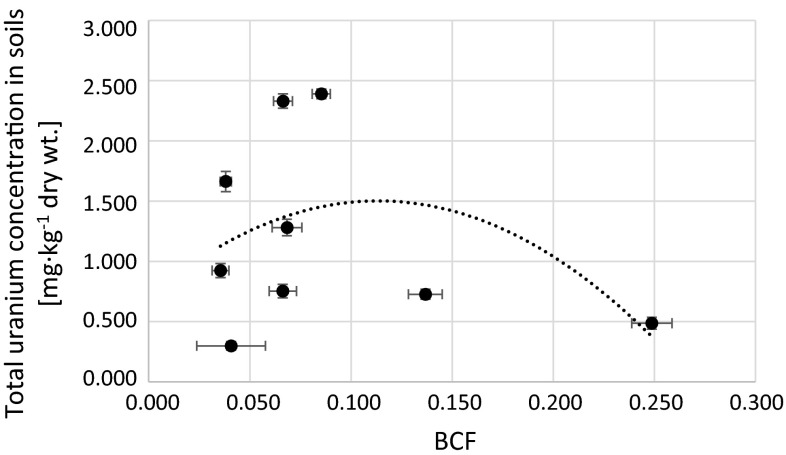
Relation between calculated BCF and total uranium concentration in soils corresponding to analyzed *Urtica dioica* plants (*r*_s_ = −0.1)

**Fig. 9:**
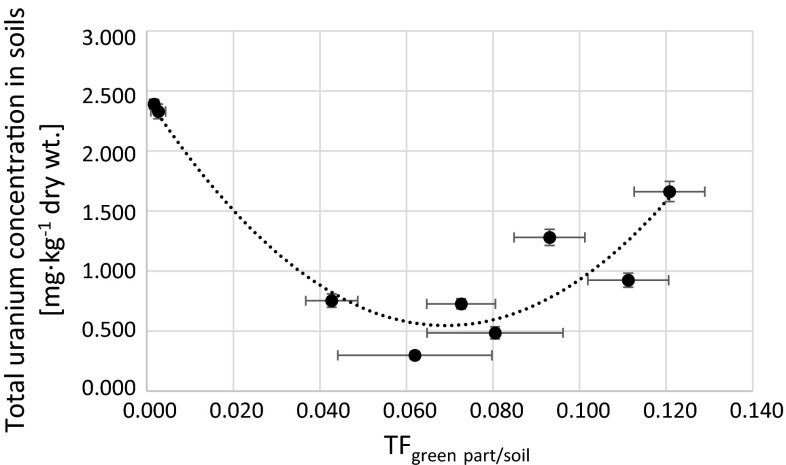
Relation between calculated TF_green part/soil_ and total uranium concentration in soils corresponding to analyzed *Urtica dioica* plants (*r*_s_ = −0.20)

**Fig. 10:**
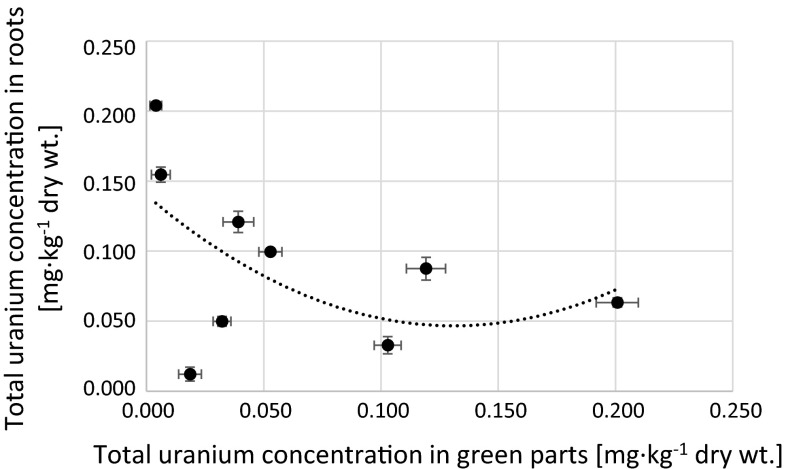
Correlation between total uranium concentration in roots and green parts of analyzed *Urtica dioica* samples (*r*_s_ = −0.40)

**Fig. 11:**
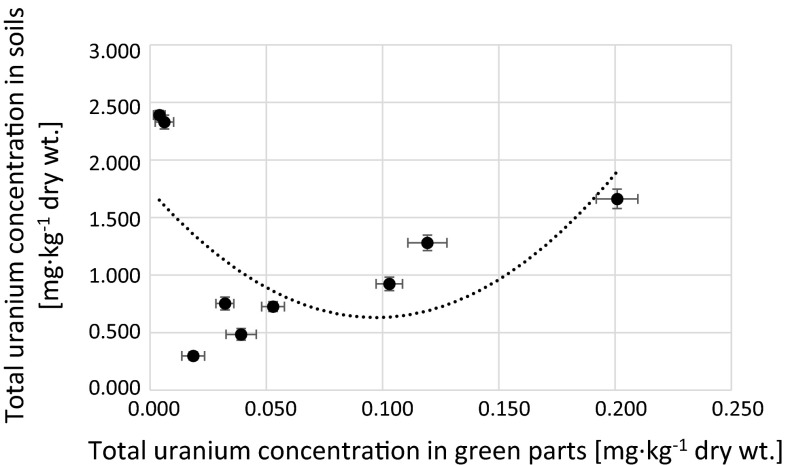
Correlation between total uranium concentration in green parts and soils corresponding to analyzed *Urtica dioica* samples (*r*_s_ = −0.11)

**Fig. 12:**
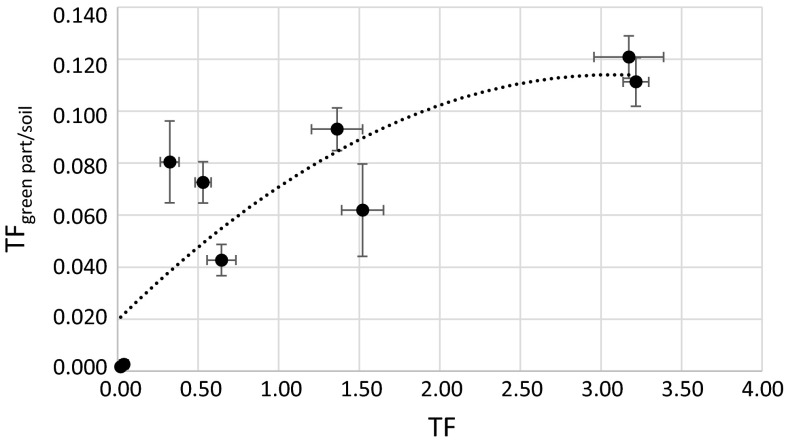
Correlation between calculated TF and TF_green part/soil_ (*r*_p_ = 0.78)

Differences on uranium uptake by plants can be explained by coil characteristics and different soils composition [46, 47]. Soil type can influence the sorption and desorption of metals. There are certain differences in bioavailability of radionuclides among soils, which may or may not be based on just quantitative properties of the soils [48]. This fact is explained by Ramaswami et al. who observed that an organic-rich soil sequestered uranium, rendering it largely unavailable for plant uptake [49]. We find weak positive correlation between uranium concentration in soils and roots (Fig. 6) and weak negative correlation between BCF_plant/soil_ (Fig. 7) and uranium concentration in soils. No correlation is observed in BCF and concentration in soils (Fig. 8). For the highest uranium concentrations in soils, although plant uptakes relatively more uranium, the BCF value is lower than for soils with less uranium. This could mean that plants may exhibit different affinities to the different uranium species. Similar effect was observed by Vandenhove et al. [47], where the uranyl cation, uranyl carbonate complexes together with the ${\text{UO}}_{ 2} {\text{PO}}_{4}^{ - }$ species, were probably the uranyl forms most readily taken up by the roots and transferred to the shoots. According to other research, plants take up radionuclides that have similar chemical behavior as the essential nutrient. Radionuclides are then transported to specific tissues based on the function of the element in plant metabolism. It is reflected in its higher concentration in a particular part compared to others [29, 40]. On the other hand, there are considerable differences in the uptake and translocation of long-lived radionuclides among different plant species [30]. These facts as well as the extent and type of plant root system and the response of plants to elements in relation to seasonal cycles might explain different TF and BCF values in plants species [21, 22]. Very often difference in uranium uptake by plants may be connected with possible air deposition. The average concentration of uranium in air close to the ground is about 0.15 ng m^−3^ and depends on the amount of suspended particles in the air [50]. Atmospheric deposition is the main source of uranium in the above-ground parts of the plants and the incorporation of the radionuclides occurs mainly from the wet deposition [9]. Lower TF_green part/soil_ might implicit that the main route of uranium accumulation in green parts is transportation via roots. Higher values suggest that air deposition is more important way. In general, roots serve as a natural barrier preventing the transport of many trace metals, including radionuclides to upper plant parts. Moreover, the radionuclide translocation from roots to shoots is probably dependent on the species [46]. The effect of soil adhesion to leaves is negligible [51]. In this study uranium concentration in soil do not affect the level of this element accumulation in *U*. *dioica* green parts (Fig. 9). There is no direct correlation between uranium concentrations in roots (Fig. 10) and soils (Fig. 11) and in green parts of analyzed plants, what is confirmed by TF values obtained for *U*. *dioica* plants. There are samples with TF values higher than one. High correlation between TF factors and TF_green part/soil_ confirms possible aerial deposition route for uranium in green parts (Fig. 12).

## Conclusions

Uranium concentration in analyzed plants and soils allows us to conclude that *U*. *dioica* is not a perfect bioindicator but it can be used as a bioimonitor of uranium contamination. Nevertheless, the level of uranium accumulation by common nettles is not extremely high. We noticed that uranium concentrations in roots depended on uranium concentrations in soils although BCF values are not correlated with its contents in soils. It suggests that different uranium species have different affinities to *U*. *dioica*. In case of distance from phosphogypsum stockpile, uranium concentration in roots and soil decreases while in green parts of some samples is high. The decrease of uranium concentration with distance for whole plants is not observed. This difference is probably connected with the fact that uranium can be uptaken by green parts from wet and dry air deposition. We can conclude that the problem of phosphogypsum stockpile is limited to the zone of maximum 300–400 m. Uranium concentrations in *U*. *dioica* samples which were collected from the slopes of the stockpile are three times higher than in plant from control area in Malbork. In case of analyzed soils it is up to six times higher. Even though we cannot neglect the fact that air deposition in the area of Wiślinka contains phosphogypsum particles.
